# *N*-Glycolylneuraminic Acid Binding of Avian and Equine H7 Influenza A Viruses

**DOI:** 10.1128/jvi.02120-21

**Published:** 2022-03-09

**Authors:** Cindy M. Spruit, Xueyong Zhu, Ilhan Tomris, María Ríos-Carrasco, Alvin X. Han, Frederik Broszeit, Roosmarijn van der Woude, Kim M. Bouwman, Michel M. T. Luu, Keita Matsuno, Yoshihiro Sakoda, Colin A. Russell, Ian A. Wilson, Geert-Jan Boons, Robert P. de Vries

**Affiliations:** a Department of Chemical Biology & Drug Discovery, Utrecht Institute for Pharmaceutical Sciences, Utrecht University, Utrecht, the Netherlands; b Department of Integrative Structural and Computational Biology, The Scripps Research Institute, La Jolla, California, USA; c Department of Medical Microbiology, Amsterdam University Medical Center, Amsterdam, the Netherlands; d Division of Risk Analysis and Management, International Institute for Zoonosis Control, Hokkaido University, Sapporo, Japan; e International Collaboration Unit, International Institute for Zoonosis Control, Hokkaido University, Sapporo, Japan; f One Health Research Center, Hokkaido University, Sapporo, Japan; g Laboratory of Microbiology, Department of Disease Control, Faculty of Veterinary Medicine, Hokkaido University, Sapporo, Japan; h Skaggs Institute for Chemical Biology, The Scripps Research Institute, La Jolla, California, USA; i Complex Carbohydrate Research Center, University of Georgia, Athens, Georgia, USA; St. Jude Children's Research Hospital

**Keywords:** glycan array, H7, hemagglutinin, *N*-glycolylneuraminic acid, NeuGc, influenza, receptor-ligand interaction, sialic acid

## Abstract

Influenza A viruses (IAV) initiate infection by binding to glycans with terminal sialic acids on the cell surface. Hosts of IAV variably express two major forms of sialic acid, *N*-acetylneuraminic acid (NeuAc) and *N*-glycolylneuraminic acid (NeuGc). NeuGc is produced in most mammals, including horses and pigs, but is absent in humans, ferrets, and birds. The only known naturally occurring IAV that exclusively bind NeuGc are extinct highly pathogenic equine H7N7 viruses. We determined the crystal structure of a representative equine H7 hemagglutinin (HA) in complex with NeuGc and observed high similarity in the receptor-binding domain with an avian H7 HA. To determine the molecular basis for NeuAc and NeuGc specificity, we performed systematic mutational analyses, based on the structural insights, on two distant avian H7 HAs and an H15 HA. We found that the A135E mutation is key for binding α2,3-linked NeuGc but does not abolish NeuAc binding. The additional mutations S128T, I130V, T189A, and K193R converted the specificity from NeuAc to NeuGc. We investigated the residues at positions 128, 130, 135, 189, and 193 in a phylogenetic analysis of avian and equine H7 HAs. This analysis revealed a clear distinction between equine and avian residues. The highest variability was observed at key position 135, of which only the equine glutamic acid led to NeuGc binding. These results demonstrate that genetically distinct H7 and H15 HAs can be switched from NeuAc to NeuGc binding and vice versa after the introduction of several mutations, providing insights into the adaptation of H7 viruses to NeuGc receptors.

**IMPORTANCE** Influenza A viruses cause millions of cases of severe illness and deaths annually. To initiate infection and replicate, the virus first needs to bind to a structure on the cell surface, like a key fitting in a lock. For influenza A viruses, these “keys” (receptors) on the cell surface are chains of sugar molecules (glycans). The terminal sugar on these glycans is often either *N*-acetylneuraminic acid (NeuAc) or *N*-glycolylneuraminic acid (NeuGc). Most influenza A viruses bind NeuAc, but a small minority bind NeuGc. NeuGc is present in species like horses, pigs, and mice but not in humans, ferrets, and birds. Here, we investigated the molecular determinants of NeuGc specificity and the origin of viruses that bind NeuGc.

## INTRODUCTION

Influenza A viruses (IAV) can infect a broad range of animals, including mammalian and avian species. Infection is initiated when the hemagglutinin (HA) on the outside of a virus particle binds to glycans with terminal sialic acid on the cell surface. The vast majority of IAV use a glycan with a terminal *N*-acetylneuraminic acid (NeuAc) as their receptor, although some strains use *N*-glycolylneuraminic acid (NeuGc) instead. Sialic acids are bound in the receptor-binding site (RBS) of the HA, consisting of conserved residues (Y98, W153, H183, and Y195) and the main structural features of the 130-loop (residues 133-139), 150-loop (residues 155-164), 190-helix (residues 190-198), and 220-loop (residues 220-229) (1). Amino acid mutations in or near the RBS can change HA binding specificity, as shown extensively for HAs binding to either α2,3-linked or α2,6-linked NeuAc (2–6).

The ability of viruses to bind either α2,3-linked or α2,6-linked sialic acids is a host determinant. Binding to either NeuAc or NeuGc could likewise affect the host range. NeuGc is present only in species that express an active form of the enzyme CMP-*N*-acetyl neuraminic acid hydroxylase (CMAH), which facilitates the hydroxylation of NeuAc to convert it to NeuGc. The gene encoding CMAH, mainly expressed in mammalian species, has been partially or completely lost at several distinct events during evolution (7), causing NeuGc to be absent in, among others, humans, ferrets, European dogs, and avian species (8–11). In species that generate NeuGc, its percentage of the total sialic acid content varies. For instance, pig trachea contains equal amounts of NeuAc and NeuGc, while 90% of the sialic acids on equine trachea and erythrocytes is NeuGc (12–15). The loss of active CMAH enzymes may have been initiated by evolutionary pressure from lethal pathogens binding to NeuGc, thereby protecting individuals with low levels of NeuGc (16). Thereupon, IAV may have coevolved with host species to bind NeuAc instead of NeuGc. Birds, which do not express NeuGc, are the reservoir for IAV.

The high NeuGc content in horses may explain why equine H7N7 viruses are the only known IAV that bind α2,3-linked NeuGc (17, 18). These highly pathogenic equine H7 viruses have not been isolated since 1978 and are, therefore, thought to be extinct (19, 20). Unlike equine H7 strains, avian and human H7 viruses, as well as related avian H15 viruses, bind NeuAc (18, 21, 22). At the moment, it is still unclear whether NeuGc could have been the archaic receptor of IAV, where the NeuGc binding of equine H7 viruses originated from, and what the molecular determinants of NeuGc specificity are.

Here, we investigated the receptor binding specificities of equine and avian H7 and H15 HAs to identify the origin of the NeuGc receptor binding of equine H7 viruses. The crystal structure of the HA of A/Equine/New York/49/73 H7N7 (H7eq) in complex with its ligand NeuGc was elucidated. Inspired by the similarities between this structure and the crystal structure of the HA of A/Turkey/Italy/214845/02 H7N3, we performed targeted mutagenesis of avian H7 and H15 HAs and the equine H7 HA. Several combinations of mutations were found that switched H7 and H15 HAs from binding NeuAc to NeuGc and vice versa. Our results demonstrate a phenotypical relationship between avian and equine H7 and H15 HA receptor binding despite the substantial genetic distance between these subtypes and provide insights into the use of NeuGc as a potentially archaic receptor for IAV.

## RESULTS

### Crystal structure of an equine H7 HA in complex with receptor analog 3′-GcLN and its similarity to an avian H7 HA.

**FIG 1:**
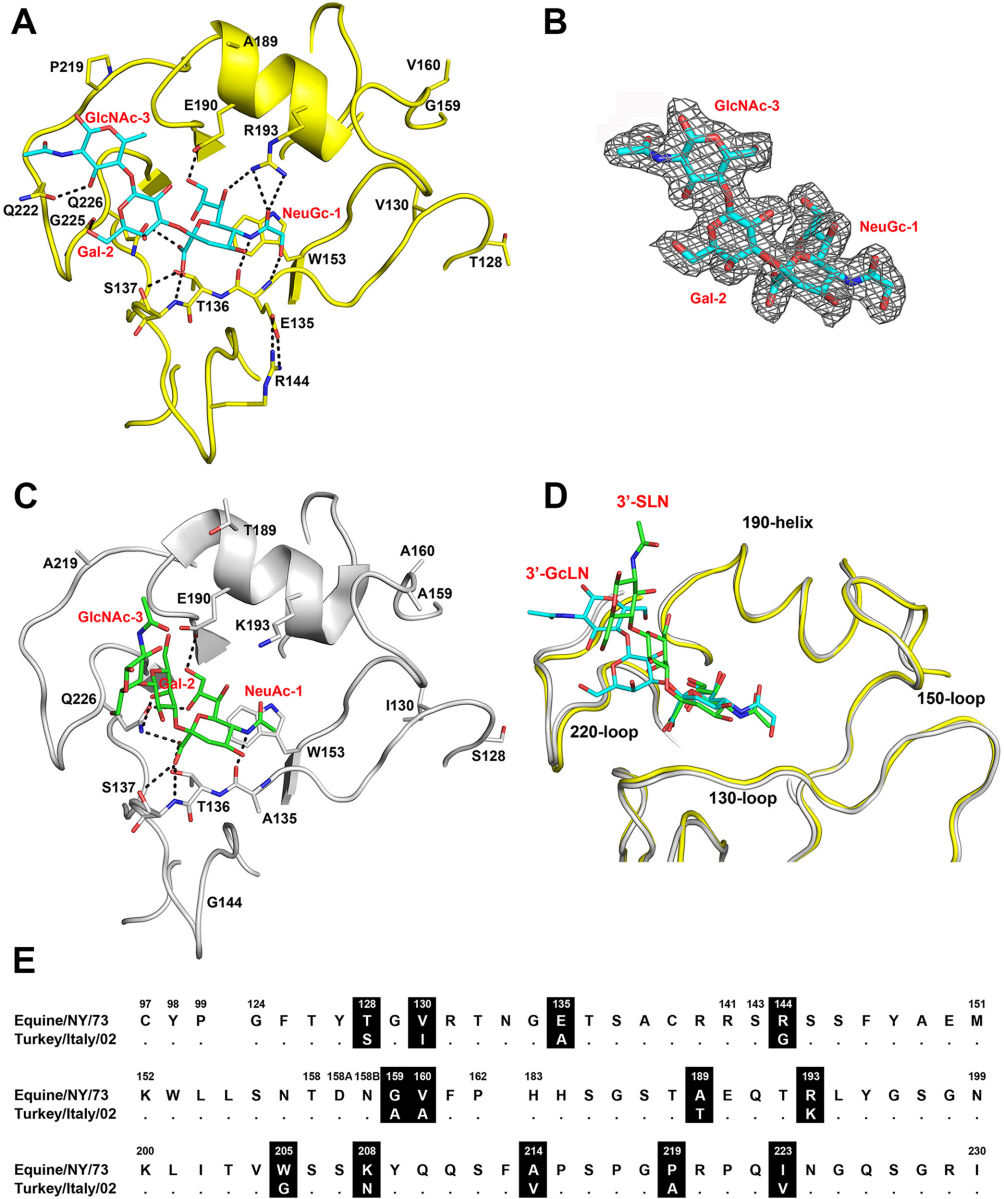
Comparison of the HAs of A/Equine/New York/43/73 H7N7 and A/Turkey/Italy/214845/02 H7N3. (A) RBS structure of H7eq (yellow) in complex with 3′-GcLN (NeuGcα2-3Galβ1-4GlcNAc; cyan), deposited in the Protein Data Bank (PDB) under accession code 7T1V. (B) Electron density 2Fo-Fc map at 1σ level for receptor analog 3′-GcLN. (C) RBS structure of H7tu (gray) in complex with 3′-SLN (NeuAcα2-3Galβ1-4GlcNAc; green) (PDB code 4BSI). (D) Superimposition of the RBS structures of H7eq and H7tu and their ligands 3′-GcLN and 3′-SLN. The coloring scheme follows that of panels A and C. (E) Alignment of the RBS residues of H7eq and H7tu, with amino acid positions (H3 numbering) indicated above the alignment, nonconserved residues highlighted in black, and dots indicating identical amino acids. A full alignment of the HAs is shown in Fig. S1.

We previously reported the crystal structure of the HA of the highly pathogenic A/Equine/New York/49/73 H7N7 (H7eq) without a ligand (PDB code 6N5A) (17). To understand the structural basis for NeuGc specificity of H7eq, we determined the crystal structure of H7eq in complex with its natural ligand 3′-GcLN (NeuGcα2-3Galβ1-4GlcNAc) at 2.05-Å resolution (Fig. 1A and Table 1) (PDB code 7T1V). The electron density for the ligand 3′-GcLN could be fitted well for all three monosaccharides (Fig. 1B). H7eq binds 3′-GcLN mainly through NeuGc-1, but the interactions extend over the 220-loop with hydrogen bonds between Gal-2 and the main-chain carbonyl oxygen of G225 and between GlcNAc-3 and the side chain of Q222 (Fig. 1A).

**TABLE 1:** Data collection and refinement statistics of H7eq in complex with 3′-GcLN

Parameter	Value^*a*^
Data collection statistics	
X-ray source	APS 23ID-D
Space group	P3
Unit cell dimensions (Å)	*a* = *b* = 112.8, *c* = 130.2
Resolution (Å)	48.87–2.05 (2.09–2.05)
No. of unique reflections	116,095 (5,442)
Redundancy	9.4 (5.2)
Avg *I*/σ(*I*)	16.1 (1.0)
Completeness	99.5 (93.2)
*R*_sym_^*b*^	0.10 (0.77)
*R*_pim_^*b*^	0.03 (0.34)
CC_1/2_^*c*^	0.99 (0.70)
No. of molecules/ASU^*d*^	3
	
Refinement statistics	
Resolution (Å)	48.87–2.05
No. of reflections in refinement	110,267
No. of refined residues	1,455
No. of refined waters	810
*R*_cryst_^*e*^	0.217
*R*_free_^*f*^	0.245
*B* values (Å^2^)	
Protein	70
RBS subdomain (residues 117–265 of HA1) of chain A, C, E	33, 40, 64
Ligand of chain A, C, E	30, 53, 100
Waters	48
Wilson *B* values (Å^2^)	28
Ramachandran values (%)^*g*^	96.3, 0
RMSD^*h*^ bond (Å)	0.009
RMSD angle (deg.)	1.43
PDB code	7T1V

a Values in parentheses are outer-shell statistics.

b *R*_sym_ = ∑*_hkl_*∑*_i_* |*I_hkl_*_,_*_i_* − <*I_hkl_*>|/∑*_hkl_*∑*_i_ I_hkl_*_,_*_i_* and *R*_pim_ = ∑*_hkl_*[1/(*N −* 1)]^1/2^ ∑*_i_* |*I_hkl_*_,_*_i_* − <*I_hkl_*>|/∑*_hkl_*∑*_i_ I_hkl_*_,_*_i_*_,_ where *I_hkl_*_,_*_i_* is the scaled intensity of the *i*th measurement of reflection *h*, *k*, *l*, < *I_hkl_*> is the average intensity for that reflection, and *N* is the redundancy. *R*_pim_ = Σ*_hkl_* [1/(*n* − 1)]^1/2^ Σ*_i_* | *I_hkl_*_,_*_i_* − *<I_hkl_>* |/Σ*_hkl_* Σ*_i_ I_hkl_*_,_*_i_*, where *n* is the redundancy.

c CC1/2 measures the Pearson correlation coefficient of one half of the data set with the second half.

d Number of molecules for complexes refers to the number of HA protomers per asymmetric unit (ASU).

e *R*_cryst_ = ∑*_hkl_* |*F_o_* – *F_c_*|/∑*_hkl_* |*F_o_*|, where *F_o_* and *F_c_* are the observed and calculated structure factors.

f *R*_free_ was calculated as for *R*_cryst_ but on 5% of data excluded before refinement.

g The values are the percentages of residues in the favored and outlier regions analyzed by MolProbity (64).

h RMSD, root mean square deviation.

The RBS structures of H7eq and the low-pathogenicity A/Turkey/Italy/214845/02 H7N3 (H7tu) (PDB code 4SBI) (23) appeared to be very similar (Fig. 1C and D), although the turkey strain binds NeuAc instead of NeuGc and was isolated almost 30 years after the equine strain. Nevertheless, 85% of the HA1 residues are identical, and the amino acid sequences around the RBS differ at 13 positions (Fig. 1E; see full alignment in Fig. S1 in the supplemental material). The NeuGc-Gal bond of 3′-GcLN in the H7eq complex adopts a *cis* conformation, which is consistent with our previous findings for the structure of 3′-GcLN in complex with the A/Vietnam/1203/2004 H5N1 Y161A mutant that shifts receptor specificity from NeuAc to NeuGc (17). In contrast, the NeuAc-Gal bond in the avian receptor analog 3′-SLN (NeuAcα2-3Galβ1-4GlcNAc) in complex with H7tu adopts a *trans* NeuAc-Gal bond (Fig. 1C and D).

In the H7eq 3′-GcLN structure, the 1-hydroxyl group of NeuGc-1 forms a hydrogen bond with the main-chain nitrogen of E135 and the E135 side chain makes a salt bridge with R144 (Fig. 1A). The amino acid at position 193 is known to be an important determinant of receptor specificity (24–27). In the complex structure of H7eq with 3′-GcLN, R193 forms a hydrogen bond with NeuGc-1 (Fig. 1A). In comparison, K193 in H7tu, with a shorter side chain, is not in hydrogen bond distance with the NeuAc-1 of 3′-SLN (Fig. 1C).

**FIG 2:**
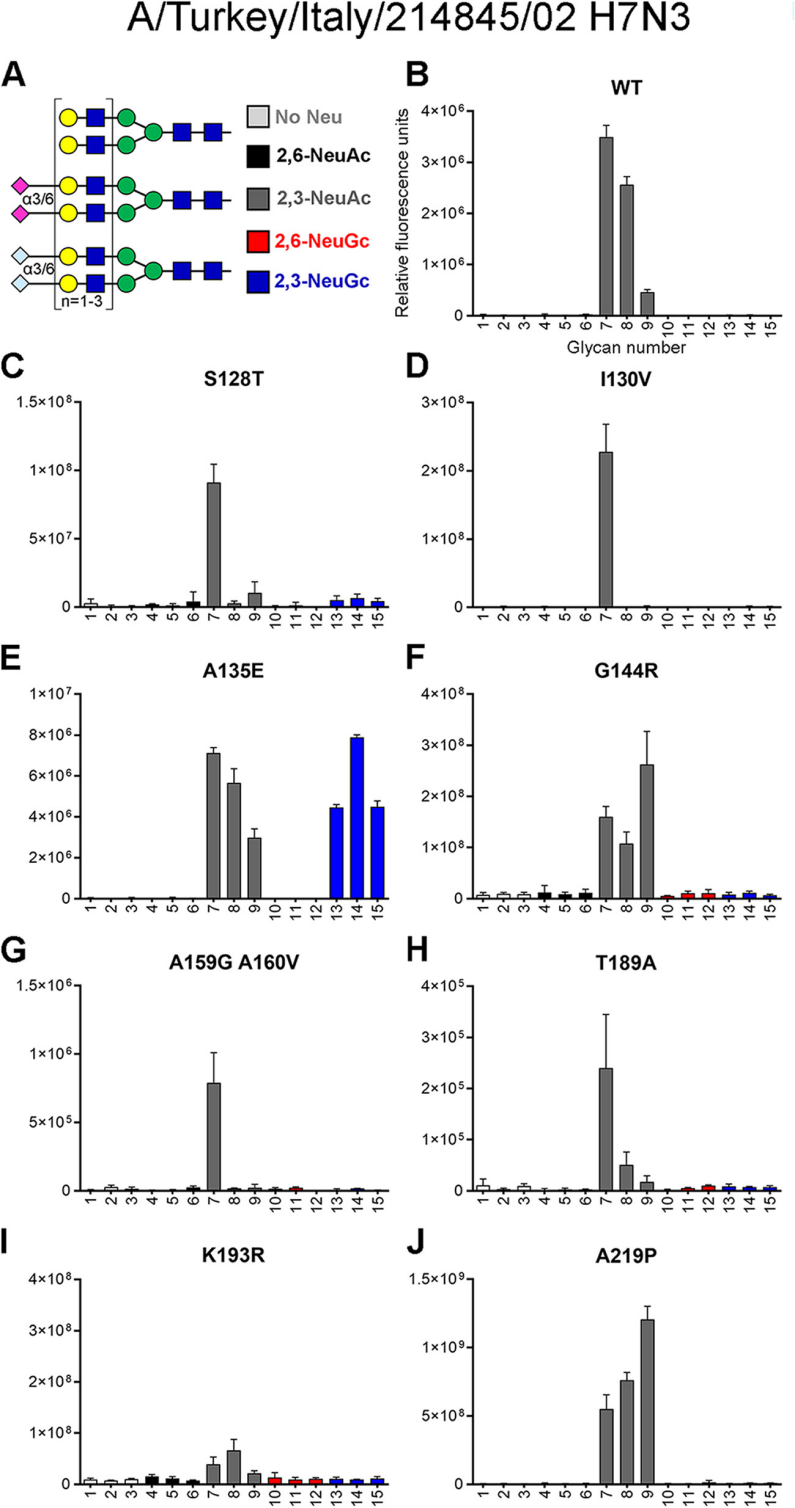
Evaluation of the binding specificities of single mutants of the HA of A/Turkey/Italy/214845/02 H7N3. (A) Synthetic glycans printed on the microarray (*n* = 6), either without sialic acid (structures 1 to 3; light gray), with α2,6-linked NeuAc (structures 4 to 6; black), α2,3-linked NeuAc (structures 7 to 9; dark gray), α2,6-linked NeuGc (structures 10 to 12; red), or α2,3-linked NeuGc (structures 13 to 15; blue). Structures 1, 4, 7, 10, and 13 contain one LacNAc repeat, structures 2, 5, 8, 11, and 14 have two repeats, and structures 3, 6, 9, 12, and 15 contain three repeats (17). The glycan microarray, which is representative of two independent assays, was used to determine the receptor specificity of recombinantly expressed HA of the H7tu wild type (WT) (B) and S128T (C), I130V (D), A135E (E), G144R (F), A159G+A160V (G), T189A (H), K193R (I), and A219P (J) mutants.

Despite these similarities in RBS structures, we found that H7tu bound solely to α2,3-linked NeuAc on the glycan array (Fig. 2B), whereas H7eq bound exclusively to α2,3-linked NeuGc (17). To decipher which residues determine NeuGc and NeuAc receptor specificity, targeted mutagenesis was performed on H7tu by replacing residues in the RBS with H7eq-like amino acids.

### Amino acid 135 is essential for binding *N*-glycolylneuraminic acid.

To identify which amino acids are critical for NeuGc binding, we mutated the HA of H7tu toward H7eq at eight locations (positions 128, 130, 135, 144, 159+160, 189, 193, and 219). Using the previously published glycan microarray containing glycans with terminal NeuAc or NeuGc (Fig. 2A) (17), we assessed the binding specificities of these recombinantly expressed HA mutants.

**FIG 3:**
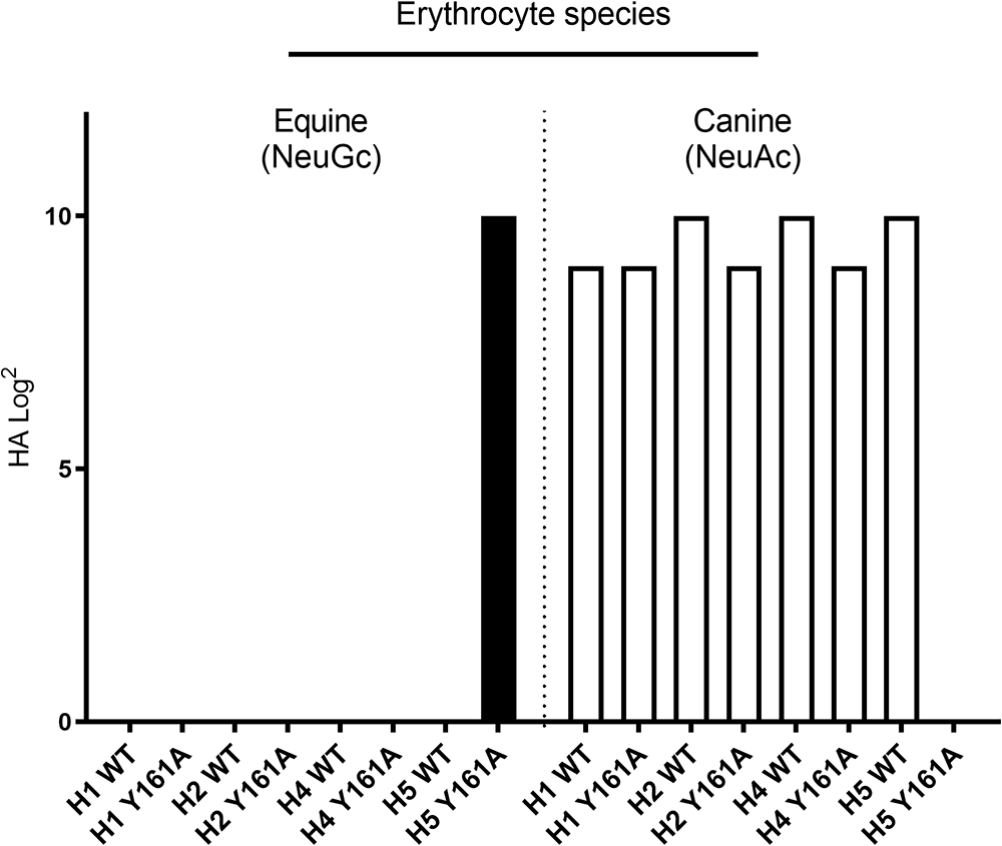
Hemagglutination assay with Y161A HA mutants on equine and canine erythrocytes. Equine erythrocytes contain approximately 90% NeuGc (12, 14, 15), and canine species cannot produce NeuGc (10). Recombinantly expressed HAs of wild-type (WT) and Y161A mutants of H1 (A/Duck/Hokkaido/111/2009 H1N5), H2 (A/Duck/Hokkaido/95/2001 H2N2), H4 (A/Duck/Hokkaido/138/2007 H4N6), and H5 (A/Vietnam/1203/2004 H5N1) were investigated.

In the 130-loop, mutations S128T and I130V did not induce clear changes in the NeuAc/NeuGc specificity (Fig. 2C and D). The amino acid at position 135 of H7 HAs is naturally diverse and has been associated with the adaptation of viruses between avian species and humans during a zoonotic outbreak of an H7N9 virus (28). We observed that mutating position 135 (A135E) resulted in a gain of binding of the H7tu HA to NeuGc while maintaining binding to NeuAc (Fig. 2E). Residue 143 has previously been suggested to be relevant for NeuGc recognition in H3 viruses (29). In H7eq, R144 forms salt bridges with the 130-loop residue E135, but mutation G144R alone in the H7tu HA did not change the binding specificity (Fig. 2F). In the 150-loop, a highly conserved tyrosine is present at position 161 in all HA subtypes except H7, H10, H12, H15, H17, and H18 (25, 30, 31). Previously, it was demonstrated that a Y161A mutation changed the binding properties of an H5 HA from NeuAc to NeuGc (17, 30). However, introducing Y161A in other HA subtypes (H1, H2, and H4) did not change binding specificity (Fig. 3). We made mutations A159G and A160V simultaneously in the H7tu HA, but unlike with the Y161A mutation in H5, we did not observe NeuGc binding with this double mutation (Fig. 2G). In the 190-helix, residue 189 is next to E190, which hydrogen bonds to the ligand, in both H7eq and H7tu (Fig. 1A and C). Mutation T189A in the H7tu HA did not change receptor specificity when introduced on its own (Fig. 2H). Residue 193 is important for ligand recognition (Fig. 1A) (24–27). Introducing K193R into the H7tu HA seemed to abolish all binding to the glycan array (Fig. 2I), even when the glycan array was illuminated with higher laser power. Despite residue 219 being very close to the 220-loop, mutation A219P did not change the binding properties of the H7tu HA from NeuAc to NeuGc (Fig. 2J). In summary, while most mutations performed on the H7tu HA did not affect binding specificity, the introduction of mutation K193R abolished glycan binding and A135E seemed to be key for binding NeuGc while maintaining binding to NeuAc.

### Various combinations of mutations switch binding from NeuAc to NeuGc.

**FIG 4:**
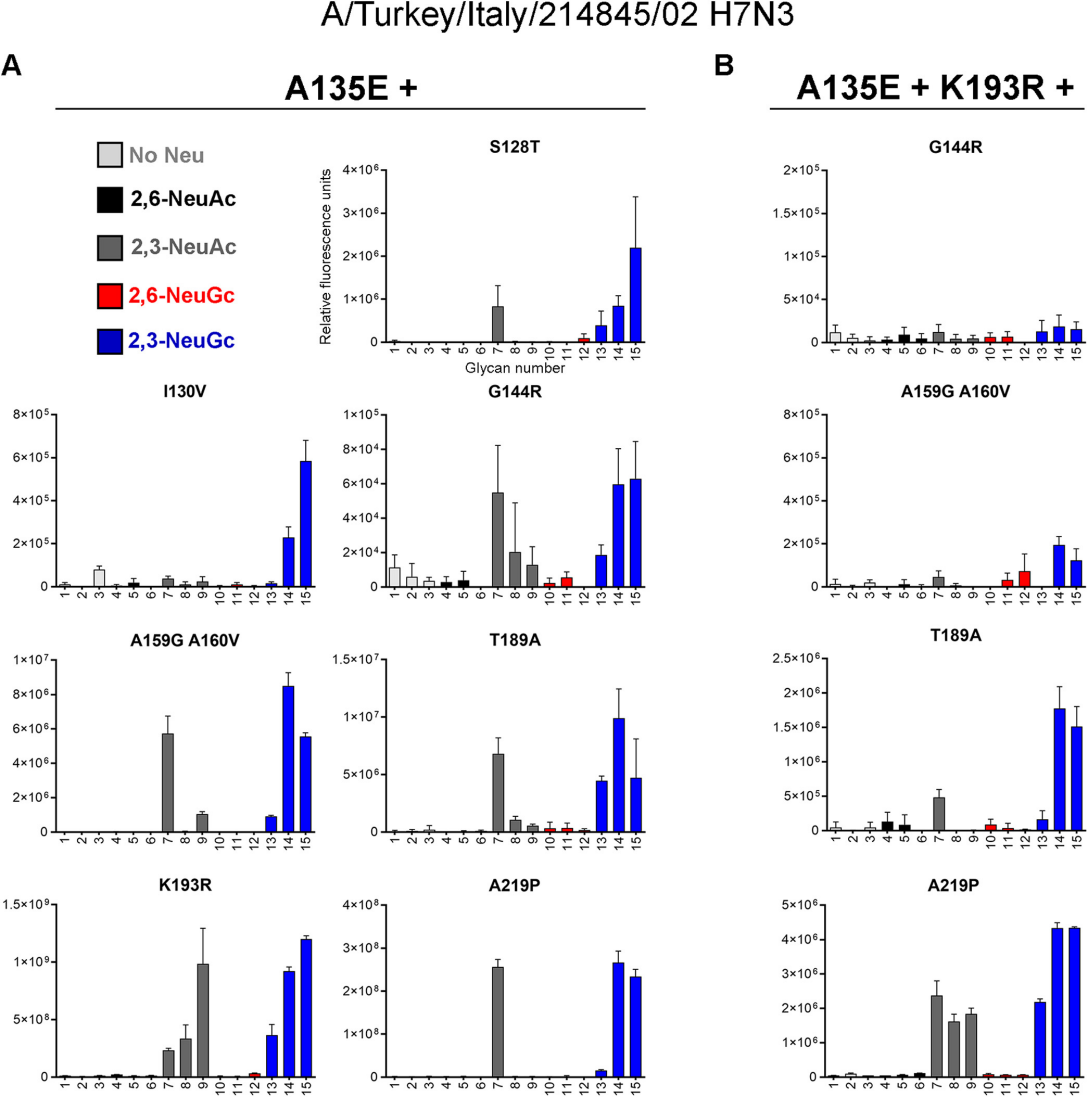
Evaluation of the binding specificities of double and triple mutants of the HA of A/Turkey/Italy/214845/02 H7N3. The glycan microarray as described in the legend to Fig. 2A was used, containing glycans with terminal NeuAc or NeuGc or without sialic acid. Representative binding specificities for two independent assays for mutant HAs containing mutation A135E and an additional mutation (S128T, I130V, G144R, A159G+A160V, T189A, K193, or A219P) (A) and mutant HAs containing mutations A135E, K193R, and an additional mutation (G144R, A159G+A160V, T189A, or A219P) (B) are shown.

Starting from the key mutation A135E, we continued mutagenesis in the recombinantly expressed HAs by adding mutations at the previously stated positions (Fig. 4A). Mutating more amino acids in the 130-loop, at position 128 (S128T) or 130 (I130V), appeared to abolish NeuAc binding while maintaining binding to NeuGc on the glycan microarray. The combination of mutation A135E with mutation G144R, A159G+A160V, T189A, or A219P did not change binding specificity compared to that of mutation A135E alone, since both NeuAc and NeuGc were still bound. Whereas almost all binding was abolished when mutation K193R was introduced by itself (Fig. 2I), the addition of mutation A135E restored binding to both NeuAc and NeuGc.

Since mutations A135E and K193R both affected the receptor-binding properties, we further combined these two mutations with mutations that did not change binding specificity so far (Fig. 4B). We found that adding mutation G144R or A159G+A160V abolished binding to the array. Adding the T189A mutation in the A135E+K193R background switched the H7tu HA to binding mainly NeuGc. The addition of mutation A219P did not affect the binding specificity, since both NeuAc and NeuGc were still bound almost equally. In short, we were able to modify the H7tu HA for binding NeuGc specifically on the glycan microarray by combinations of mutations A135E+S128T, A135E+I130V, or A135E+T189A+K193R. These results show that residues in the 130-loop or 190-helix modify the specificity toward NeuGc.

### No binding specificity to avian or equine erythrocytes or tracheal epithelium was observed for avian H7 mutants that bind NeuGc on the glycan microarray.

A glycan microarray, as used in this study, is a sophisticated tool to investigate the binding of proteins to synthetic glycans of which we know the exact structure. However, not all natural host glycans can be present on the array, and therefore, it is necessary to investigate the binding specificities of HAs to host cells and tissues. Avian species lack a functional CMAH and therefore do not have NeuGc. In contrast, on equine erythrocytes and tracheal tissue, approximately 90% of the sialic acids are NeuGc (12, 14, 15), and to our knowledge, there are no species with a higher percentage of NeuGc. Therefore, we performed a hemagglutination assay with avian and equine erythrocytes and tissue staining on tracheal epithelium, which is the natural location of infection, of the same species.

As controls for the presence of NeuAc and NeuGc on erythrocytes and tracheal epithelium, we used our previously studied wild-type (WT) and Y161A mutant HAs of A/Vietnam/1203/2004 H5N1 (H5VN), which specifically bind α2,3-linked NeuAc and α2,3-linked NeuGc, respectively. To our knowledge, these HAs are the only available controls for specific NeuAc and NeuGc binding (17). The virus particles from these viruses, however, do not show exclusive specificity for NeuAc or NeuGc (17) and are therefore not appropriate to show the presence of these sialic acids.

**FIG 5:**
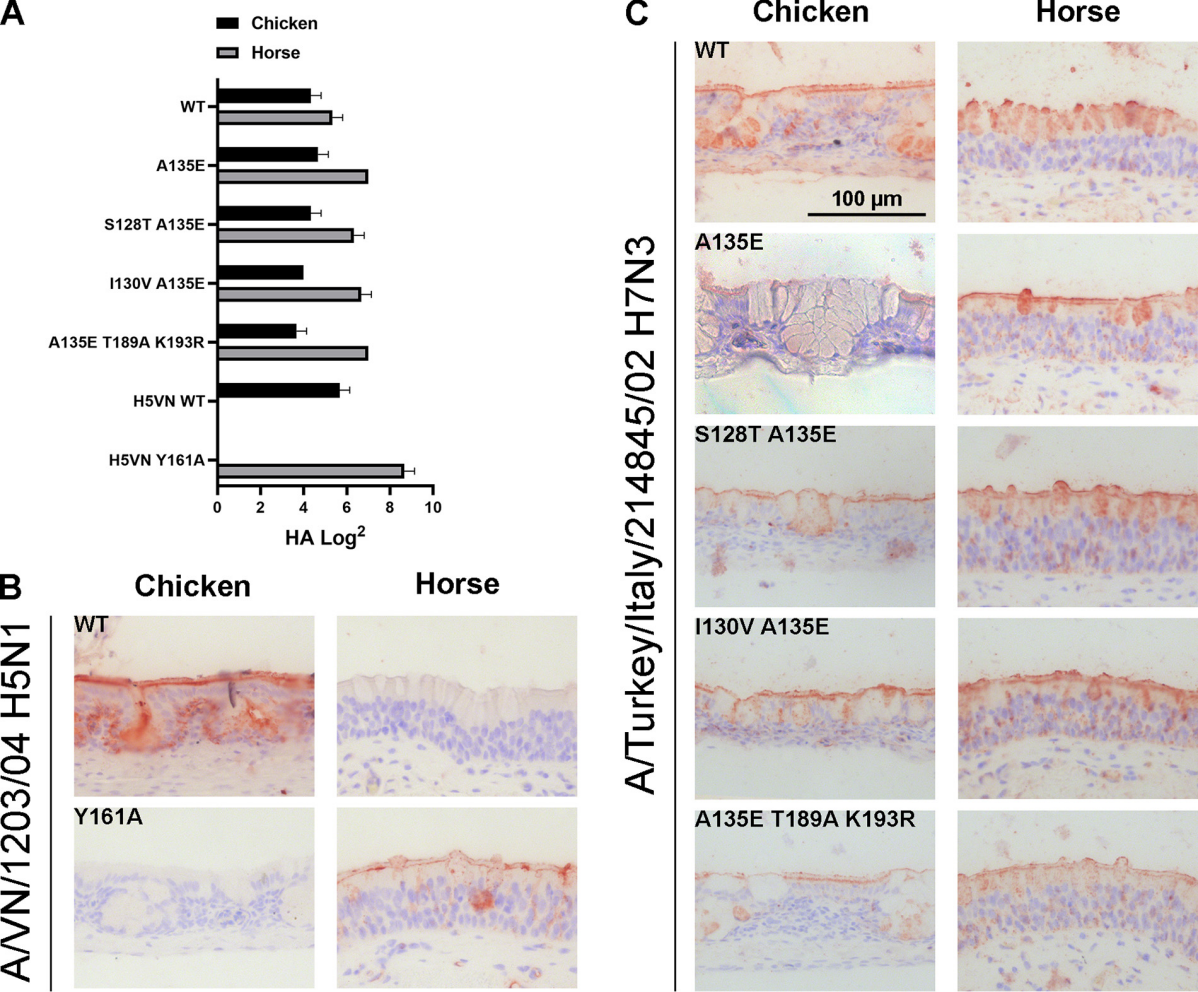
Binding specificities of (mutant) HA of A/Turkey/Italy/214845/02 H7N3 to chicken and horse erythrocytes and tracheal epithelium. (A) A hemagglutination assay (*n* = 3, mean + standard deviation [SD] shown) with chicken and horse erythrocytes was performed using H7tu WT and mutant HAs (A135E, A135E+S128T, A135E+I130V, and A135E+T189A+K193R). AEC staining was used to visualize tissue binding. Tissue staining of chicken and horse tracheal epithelium was performed with WT and Y161A mutant HA of A/Vietnam/1203/2004 H5N1 as a positive and negative control (B) and H7tu WT or mutant HAs as described for panel A (C).

While binding only to NeuAc on the glycan array, the WT H7tu HA agglutinated both chicken erythrocytes, which contain only NeuAc (7, 8), and horse erythrocytes, which contain mainly NeuGc and a small portion of NeuAc (12, 14, 15) (Fig. 5A). Therefore, a loss of binding to chicken erythrocytes would indicate a loss of NeuAc binding. However, both types of erythrocytes were still bound by HAs with combinations of all investigated mutations (A135E, A135E+S128T, A135E+I130V, and A135E+T189A+K193R) (Fig. 5A), and therefore, no conclusions concerning NeuGc specificity could be drawn from these hemagglutination experiments. Similarly, the WT and all mutants of H7tu bound both horse and chicken tracheal tissue (Fig. 5C). The results demonstrated that there are some differences in glycan binding between the glycan array, hemagglutination assay, and tissue staining.

### NeuGc binding specificity can also be achieved in another avian H7 strain.

**FIG 6:**
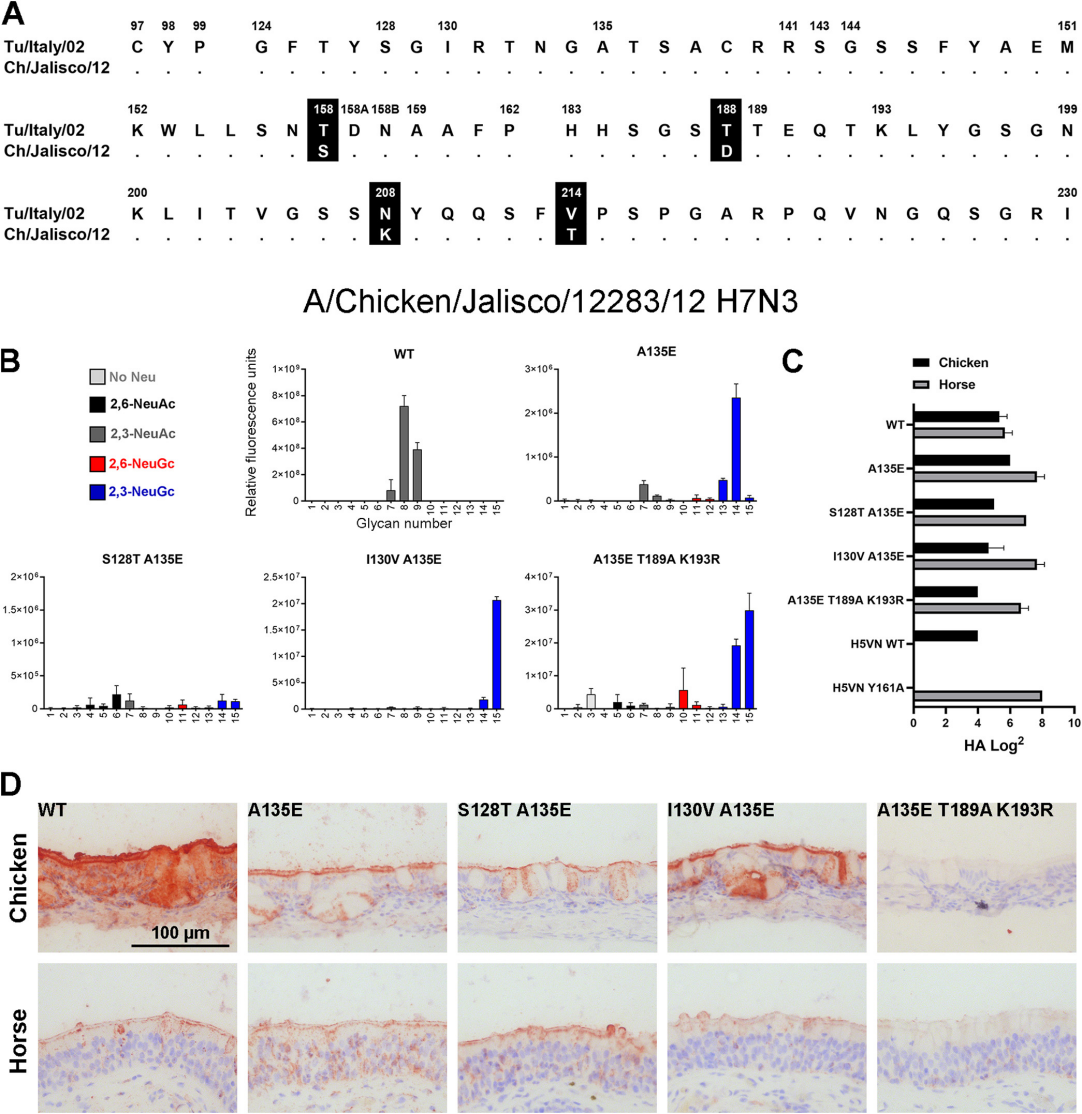
Evaluation of the binding specificities of the (mutant) HA of A/Chicken/Jalisco/12283/12 H7N3. (A) Alignment of the RBS of the HAs of A/Turkey/Italy/214845/02 H7N3 and A/Chicken/Jalisco/12283/12 H7N3, with the amino acid positions indicated above the alignment and dots indicating identical amino acids. A full alignment of the HAs is shown in Fig. S1. (B) The binding specificities of WT HA of A/Chicken/Jalisco/12283/12 H7N3 and A135E, S128T+A135E, I130V+A135E, and A135E+T189A+K193R mutant HAs were evaluated on the glycan microarray as described in the legend to Fig. 2A. (C) Binding specificities of WT and mutant HAs were further tested in a hemagglutination assay on chicken and horse erythrocytes (*n* = 3, mean + SD shown). (D) The binding of the WT and mutant HAs to chicken and horse tracheal epithelium (controls shown in Fig. 5B) is visualized using AEC staining.

To investigate whether the mutations that were found to switch the HA of A/Turkey/Italy/214845/02 H7N3 (a virus from the Eurasian lineage) toward NeuGc binding are universal among H7 strains, we analyzed the HA of another avian strain from the North American lineage, the A/Chicken/Jalisco/12283/2012 H7N3, which is highly pathogenic. Alignment of the HA sequences showed that the two strains differ at four amino acid positions (158, 188, 208, and 214) in the otherwise very similar RBS (Fig. 6A, see full alignment in Fig. S1 in the supplemental material). In the glycan array analysis, the WT HA of A/Chicken/Jalisco/12283/12 H7N3 bound NeuAc (Fig. 6B). The sole introduction of mutation A135E enabled NeuGc binding and seemed to abolish some binding to NeuAc. Furthermore, NeuGc binding specificity on the glycan array was achieved by combining mutation A135E with mutations I130V or T189A+K193R. A combination of A135E and S128T resulted in a loss of glycan binding on the array.

The combinations of mutations A135E, A135E+S128T, and A135E+I130V did not change the binding specificity of the HA in the hemagglutination assay using chicken or horse erythrocytes (Fig. 6C), or on equine and avian trachea (Fig. 6D), as both species were still bound, similar to observations in H7tu. The combination of mutations A135E, T189A, and K193R did not change the binding specificity in the hemagglutination assay either, but binding to both chicken and horse tracheal tissue was lost. Nevertheless, based on the glycan array analysis, we conclude that distant avian H7 HAs from different lineages can acquire NeuGc binding through identical amino acid changes.

### Reciprocal mutations in the equine H7 HA allow binding to NeuAc.

**FIG 7:**
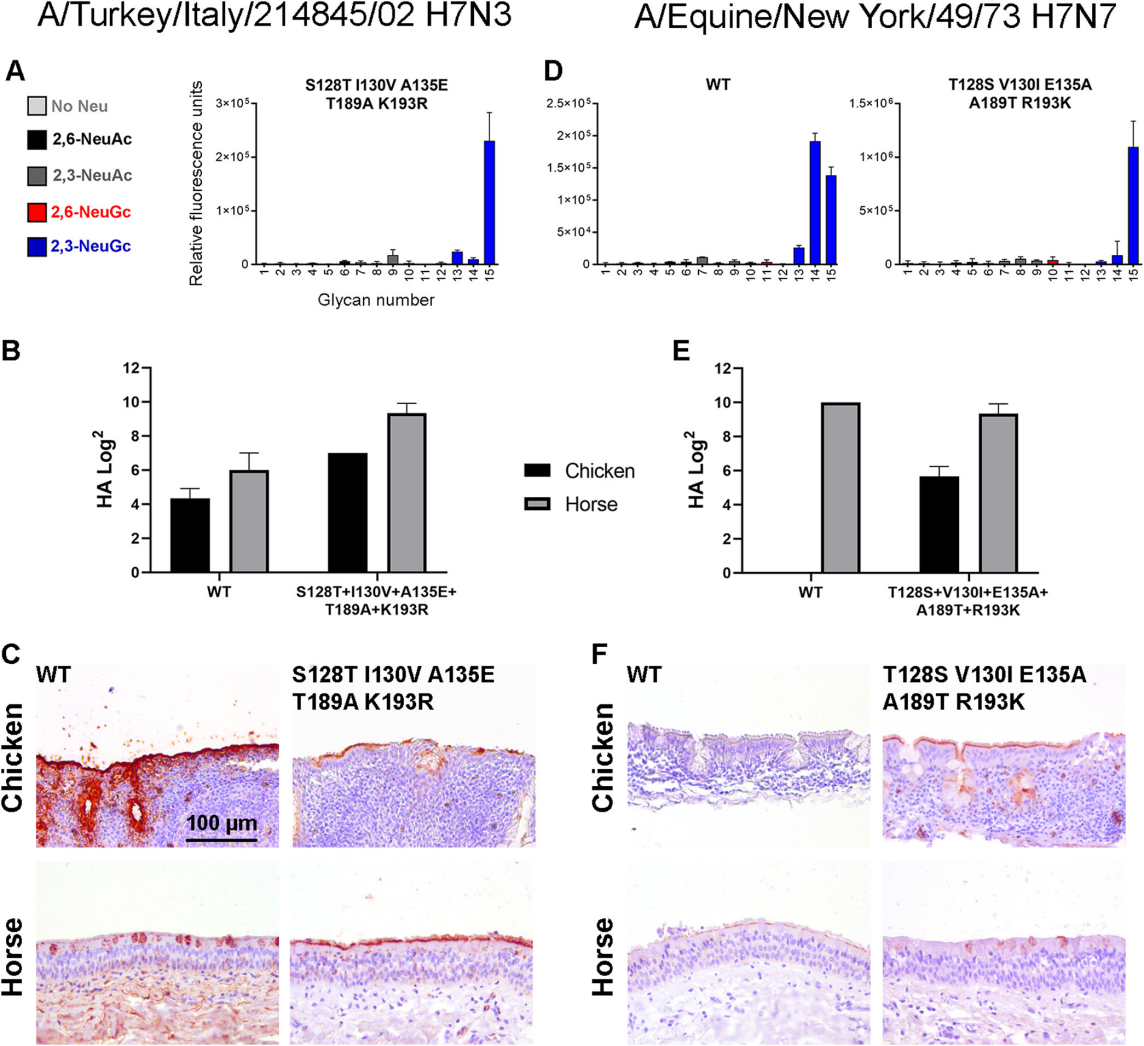
Effect of the combination of the 5 mutations at positions 128, 130, 135, 189, and 193 in A/Turkey/Italy/214845/02 H7N3 and A/Equine/New York/43/73 H7N7 HA. (A to C) The binding specificity of the mutant HA (S128T, I130V, A135E, T189A, K193R) of A/Turkey/Italy/214845/02 H7N3 was evaluated by using the glycan microarray as described in the legend to Fig. 2A (A), by the hemagglutination assay with chicken and horse erythrocytes (*n* = 3, mean and SD shown) (with H5 NeuAc and NeuGc controls shown in Fig. 9) (B), and by immunohistochemistry using chicken and horse tracheal epithelium (controls shown in Fig. 5B), visualized using AEC staining (C). (D to F) Likewise, the binding specificities of the WT and mutant (T128S, V130I, E135A, A189T, R189K) HAs of A/Equine/New York/43/73 H7N7 were evaluated by using the glycan microarray in which the WT had been previously investigated (17) (D), the hemagglutination assay (E), and tissue staining (F).

Since we did not observe exclusive NeuGc binding with the double and triple mutants, we combined the five mutations S128T, I130V, A135E, T189A, and K193R in the H7tu HA. With this mutant, similar results were achieved as previously, with NeuGc binding on the glycan array (Fig. 7A) and binding to both horse and chicken erythrocytes (Fig. 7B) and tracheal epithelium (Fig. 7C).

The WT H7eq HA was previously shown to bind NeuGc on the glycan array (17), and here we showed that this WT HA also specifically bound horse erythrocytes (Fig. 7E) and tracheal epithelium (Fig. 7F). After the introduction of the five reciprocal mutations (T128S, V130I, E135A, A189T, R189K), the equine HA still seemed specific for NeuGc on the glycan array (Fig. 7D) but started to bind to chicken erythrocytes and tracheal epithelium, while binding to equine tracheal epithelium was decreased. Since chickens are unable to produce NeuGc, this switch in binding indicated that the mutant H7Eq HA gained the ability to bind to NeuAc.

### Equine and avian H7 strains are evolutionarily distant.

**FIG 8:**
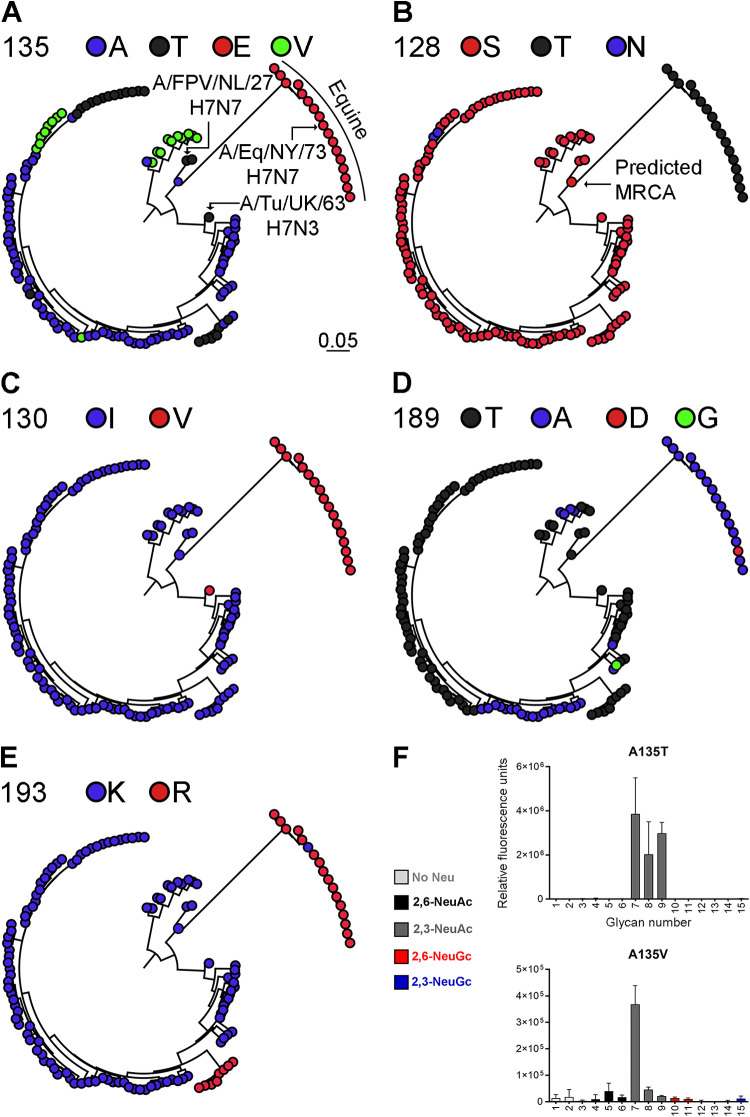
Phylogenetic tree of equine and avian H7 HA sequences and evaluation of the binding specificities of mutants of the HA of A/Turkey/Italy/214845/02 H7N3 at amino acid position 135. Phylogenetic trees of equine and avian H7 IAV strains from the Eurasian lineage were reconstructed. The equine H7 strains cluster as a single monophyletic clade. The avian strains that are most closely related to the equine strains (A/FPV/Dutch/1927 H7N7 and A/Turkey/England/1963 H7N3) are indicated, as well as A/Equine/New York/43/73 H7N7. The annotated phylogenetic tree with all strain names is shown in Fig. S2. The variation in amino acids at positions 135 (alanine, threonine, glutamic acid, valine) (A), 128 (serine, threonine, asparagine) (B), 130 (isoleucine, valine) (C), 189 (threonine, alanine, aspartic acid, glycine) (D), and 193 (lysine, arginine) (E) is shown. For all positions, the amino acid of the predicted most recent common ancestor (MRCA) is shown. (F) Representative binding specificities on the glycan microarray (as described in the legend to Fig. 2A) for H7tu A135T and A135V mutants are shown.

The fact that avian H7 HAs can be mutated toward binding NeuGc and equine H7 HAs can be mutated toward binding NeuAc suggests that equine and avian H7 strains are phenotypically related. To investigate the genetic relationship between H7 strains, we reconstructed a maximum likelihood (ML) phylogenetic tree using HA sequences of equine H7 strains and their most closely related Eurasian avian H7 strains (Fig. 8A to E; Fig. S3). All equine strains cluster under a single monophyletic clade. Strains A/FPV/Dutch/1927 H7N7 and A/Fowl/Weybridge/1934 H7N7 appeared to be the most closely related avian strains to the equine viruses. We investigated the natural variation in amino acids at positions for which binding specificity changed (128, 130, 135, 189, and 193).

For each selected amino acid position, we annotated the ML tree based on the variation in residues (Fig. 8A to E). The predicted most recent common ancestor (MRCA) at all positions contained avian-like amino acids. Of the five investigated amino acid positions, the highest variability in amino acids is present at key position 135, although we observed a clear distinction between a glutamic acid in the equine strains and a variation of alanine, valine, and threonine in the avian strains (Fig. 8A). At position 128, there is an obvious distinction between the threonine in equine strains and mainly serine in the avian strains (Fig. 8B). Again, a clear difference was observed at position 130 between avian strains (isoleucine) and equine strains (valine). Surprisingly, a closely related avian strain, A/Turkey/England/1963 H7N3, also contains a valine at position 130, just like the equine strains (Fig. 8C). At position 189, all but one of the equine strains contain an alanine, while there is a variety of mainly alanine and threonine present in the avian strains (Fig. 8D). At position 193, nearly all equine strains contain an arginine, whereas most avian strains, except for a small clade of viruses from chickens in Pakistan, contain a lysine (Fig. 8E). We conclude that there is a clear distinction between the amino acids in the equine and avian strains, with the highest variability in residues being present at position 135, which we investigated further using targeted mutagenesis.

Four different amino acids (glutamic acid, alanine, valine, and threonine) are naturally present at position 135 of avian and equine H7 viruses (Fig. 8A). When the alanine is encoded by either GCG or GCA, changing a single base pair will change the amino acid to glutamic acid, valine, or threonine. We introduced all these residues at position 135 of H7tu to investigate whether the acquisition of NeuGc binding was specific for the glutamic acid. This was indeed the case, as the introduction of a threonine or a valine at position 135 did not promote binding to NeuGc (Fig. 8F).

### H15 HA can also be switched to NeuGc binding.

**FIG 9:**
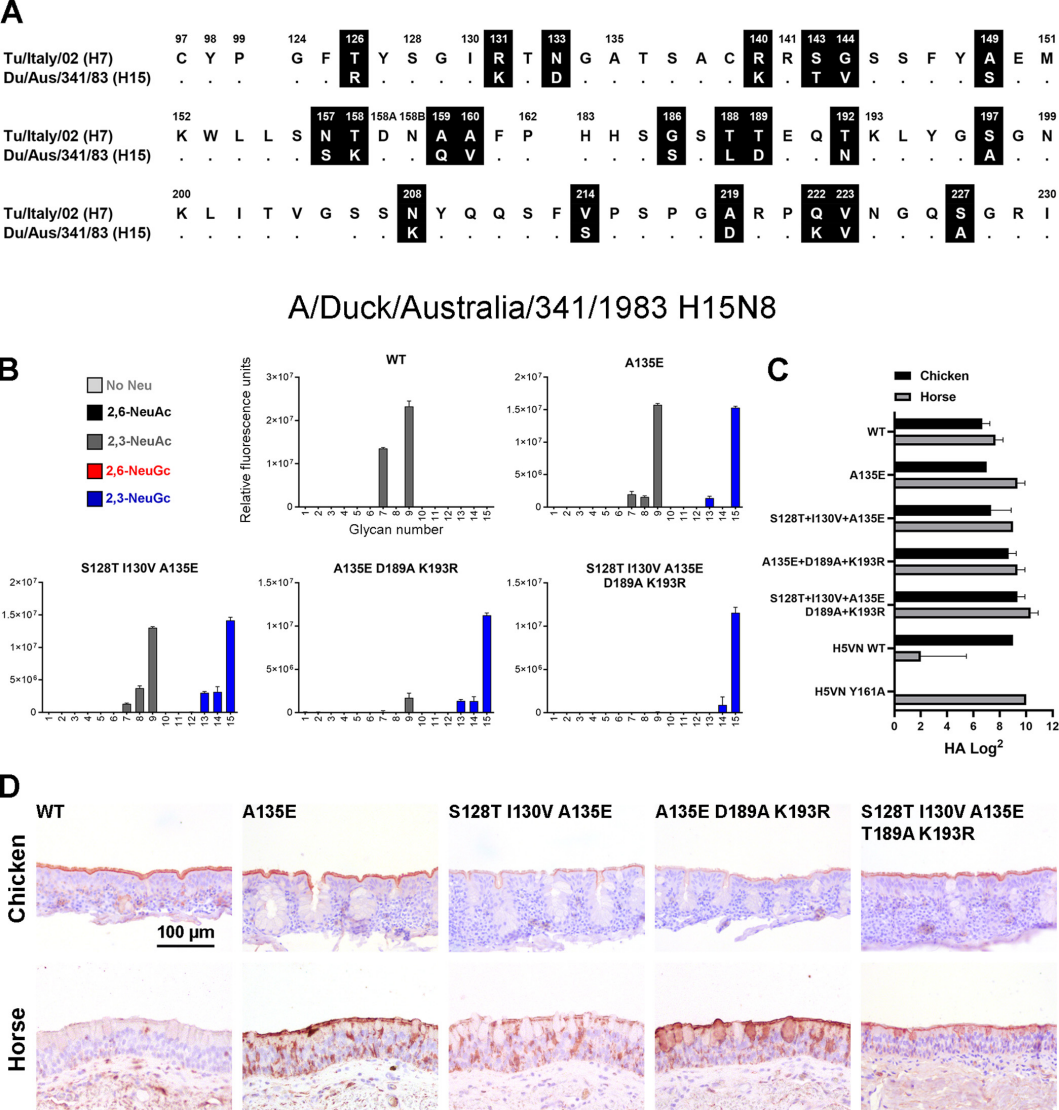
Evaluation of the binding specificities of the (mutant) HA of A/Duck/Australia/341/1983 H15N8. (A) Alignment of the RBS of the HAs of A/Turkey/Italy/214845/02 H7N3 and A/Duck/Australia/341/1983 H15N8, with the amino acid positions indicated above the alignment and dots indicating identical amino acids. A full alignment of the HAs is shown in Fig. S1. (B) The binding specificities of WT HA of A/Duck/Australia/341/1983 H15N8 and A135E, S128T+I130V+A135E, A135E+T189A+K193R, and S128T+I130V+A135E+T189A+K193R mutant Has were evaluated on the glycan microarray as described in the legend to Fig. 2A. (C) Binding specificities of WT and mutant HAs were further tested in a hemagglutination assay on chicken and horse erythrocytes (*n* = 3, mean + SD shown). (D) Binding of the WT and mutant HAs to chicken and horse tracheal epithelium (controls shown in Fig. 5B) is visualized using AEC staining.

To further investigate the conservation of the switch to NeuGc binding due to mutations at positions 128, 130, 135, 189, and 193 in other subtypes, we investigated the receptor binding of the HA of the low-pathogenicity A/Duck/Australia/341/1983 H15N8 virus. H15 and H7 viruses are related and are present in one subgroup together with H10 viruses (21). Nevertheless, there are 21 amino acid differences between the RBS of H7tu and this H15 (Fig. 9A; full alignment in Fig. S1), which is much more than the four different residues between the two distant avian H7 strains that we investigated (Fig. 6A).

As with other avian H15 viruses (22, 32), we found that this WT H15 HA also bound α2,3-linked NeuAc on the glycan array (Fig. 9B). Furthermore, the WT H15 HA bound both horse and chicken erythrocytes in the hemagglutination assay (Fig. 9C), as was also observed for the two studied avian H7 HAs. However, the WT H15 HA bound only chicken, not horse, tracheal epithelium (Fig. 9D), which is different from the investigated avian H7 HAs.

As soon as the key mutation A135E was introduced, the HA showed similar binding patterns as the avian H7 Has, with binding to both NeuAc and NeuGc and both chicken and horse erythrocytes and tissue (Fig. 9B to D). The combination of A135E+S128T+I130V did not change the binding specificities in comparison to that of only A135E. When we, however, combined mutations A135E, D189A, and K193R or all five mutations, the H15 HA became specific for NeuGc on the glycan array. As with the avian H7 HAs, this was not observed in the hemagglutination assay and tissue staining.

## DISCUSSION

To elucidate the molecular determinants for NeuGc binding, we determined the crystal structure of the HA of A/Equine/New York/49/73 H7N7 in complex with its receptor analog 3′-GcLN (NeuGcα2-3Galβ1-4GlcNAc). The overall RBS structures of H7eq and A/Turkey/Italy/214845/02 H7N3 were shown to be similar. To examine the critical amino acids for NeuGc binding, we performed mutational analysis on two distant avian H7 HAs and an avian H15 HA that specifically bound NeuAc. Previously, we demonstrated that HAs can bind either NeuAc or NeuGc (17). Here, we demonstrate that avian H7 and H15 HAs can bind both NeuAc and NeuGc by the introduction of A135E.

We previously studied the NeuAc-specific HA of A/Vietnam/1203/2004 H5N1 (H5VN) and its Y161A mutant that is specific for NeuGc, which showed complete specificity on the glycan microarray, in the hemagglutination assay (17), and on tracheal epithelium tissue (Fig. 5C). Similarly, we found that WT H1, H2, and H4 HAs bind dog, but not horse, erythrocytes in the hemagglutination assay (Fig. 3). In contrast, we observed that WT avian H7 HAs bound both chicken (NeuAc) (7, 8) and horse (mainly NeuGc) (12, 14, 15) erythrocytes and tracheal tissue while binding specifically to NeuAc on the glycan microarray. These findings then distinguish these H7 viruses from other subtypes of IAV. Possibly, the presence of NeuAc on horse erythrocytes and tracheal tissue, although estimated to be less than 10% of the total sialic acids (12, 14, 15), was sufficient to be bound by the WT avian H7 HAs. Additionally, the residual NeuAc-binding capacity of the mutant avian H7 HAs may explain the binding to chicken erythrocytes and tissue. Importantly, not all compounds that are naturally present in the host are represented on the glycan microarray. Therefore, missing glycans on the array may explain the binding of avian H7 HAs to horse and chicken erythrocytes and tissue.

The natural variety in glycans in nature is massive, and it is impossible to synthesize all of these glycans for our glycan microarray. For example, glycans can be elongated, either symmetrically or asymmetrically, be tri- or tetra-antennary, and contain one or multiple terminal sialic acids. Furthermore, the addition of fucose at different positions on LacNAc structures gives rise to different Lewis antigens, and additional sulfate or O-acetyl groups can be present, adding another layer of complexity. Fucosylated (Lewis X) and sulfated glycans are present in the human lung (33, 34), and sulfated glycans have been observed in porcine lungs (35). For equine and avian species, the glycans in the respiratory tract have not been studied in detail yet. One study describes the presence of sialyl Lewis X structures in the respiratory tract of chickens (36). Little is known about the glycans present on erythrocytes of different species, apart from two studies that describe that very few glycans with fucoses are present on chicken and mouse erythrocytes (37, 38). IAV of different subtypes (H1, H3, H4, H5, H6, H7, H9, H13, H14) that (specifically) bind, or do not bind at all, to fucosylated and sulfated glycans are known (39–46). Most relevant, avian, human, and seal H7 HAs also prefer to bind sulfated sialyl Lewis X structures (18, 40, 43). In conclusion, fine receptor binding specificities regarding fucosylation and sulfation, which are observed in many different IAV, may be present for the avian and equine H7 and H15 HAs besides the NeuAc and NeuGc that are studied here.

It has been suggested that recognition of NeuGc by IAV is essential for viral replication in horses (12). The most prevalent IAV among horses currently and in the past are H3N8 and H7N7 viruses (47). While equine H7N7 viruses have been shown to prefer binding to NeuGc (17, 18), the currently circulating equine H3N8 viruses bind to NeuAc (18), indicating that these viruses may not be under pressure to adapt to NeuGc binding since still small amounts of NeuAc are present in horses. These equine H3N8 viruses often infect dogs as well (47, 48), which are not able to make glycans containing NeuGc due to the lack of a functional CMAH. Furthermore, NeuAc binding could be advantageous for IAV to maintain circulation in NeuAc-rich reservoirs. Additionally, if NeuGc binding were required for replication of IAV in horses, more equine IAV that bind NeuGc would be expected. Therefore, it seems unlikely that NeuGc recognition by IAV is essential for replication in horses.

Equine and avian H7 strains are estimated, by phylogenetic analysis, to have separated in the mid- to late 1800s, and separation between H7 and H15 viruses is estimated to have taken place in the early 1800s (49). Nevertheless, here we demonstrated that avian H7 and H15 HAs, although genetically distinct from equine H7 viruses, are able to bind NeuGc after the introduction of mutation A135E. Further NeuGc specificity was obtained when mutations were added at positions 128, 130, 189, and 193. Reciprocal mutations in an equine H7 HA likewise resulted in the ability to bind NeuAc. These findings suggest that avian and horse H7 and H15 IAV are phenotypically related.

We further showed that a broad range of IAV can bind NeuGc with the introduction of a few mutations. Furthermore, CMAH genes have been inactivated at several distinct events in evolution (7), causing the loss of NeuGc expression in different species over time. This loss of CMAH activity was potentially triggered by evolutionary pressure from lethal pathogens binding to NeuGc (16). Therefore, we have taken the opportunity to consider NeuGc as a potential archaic receptor of IAV.

## MATERIALS AND METHODS

### Expression, crystallization, and structural determination of the equine H7 HA in complex with receptor analog 3′-GcLN.

The HA ectodomain of A/Equine/New York/49/73 H7N7 (GenBank accession no. LC414434) was cloned and expressed as described previously (17). Briefly, cDNA corresponding to residues 11 to 327 of HA1 and 1 to 179 of HA2 (H3 numbering) was cloned into a pFastbac vector. The HA was expressed in Hi5 insect cells as described previously (50), after which it was purified, the trimerization domain and His_6_ tag were removed, and the HA was concentrated to 6 mg/mL in 20 mM Tris, pH 8.0, 150 mM NaCl.

Crystals of the H7eq HA were obtained at 20°C using the vapor diffusion sitting drop method against a reservoir solution containing 32% (wt/vol) polyethylene glycol 400 and 0.1 M CAPS (*N*-cyclohexyl-3-aminopropanesulfonic acid) at pH 10. The complex of HA protein with 3′-GcLN was obtained by soaking HA crystals in a reservoir that contained 3′-GcLN to a final concentration of 10 mM for 1 h at 20°C. The crystals were flash cooled in liquid nitrogen, without additional cryoprotectant, before X-ray data collection at the Advanced Photon Source (APS) (Table 1). Data integration and scaling were performed using HKL2000 (51). Molecular replacement using Phaser (52) was used to solve the H7eq complex structure, for which an apo H7eq HA structure (PDB code 6N5A) was utilized as the search model. REFMAC5 (53) was used for structure refinement, and modeling was done with COOT (54). The final refinement statistics are outlined in Table 1.

### Expression and purification of HA for binding studies.

HA-encoding cDNAs of A/Turkey/Italy/214845/02 H7N3 (23) (synthesized and codon optimized by GenScript), A/Chicken/Jalisco/12283/12 H7N3 (a kind gift from Florian Krammer, Mt. Sinai Medical School), A/Duck/Australia/341/1983 H15N8 (a kind gift from Keita Matsuno), A/Equine/New York/49/73 H7N7 (a kind gift from Keita Matsuno), and A/Vietnam/1203/2004 H5N1 (synthesized and codon optimized by GenScript) were cloned into the pCD5 expression vector as described previously (55, 56). The pCD5 expression vector was adapted to clone the HA-encoding cDNAs in frame with DNA sequences coding for a secretion signal sequence, the Twin-Strep (WSHPQFEKGGGSGGGSWSHPQFEK; IBA, Germany), a GCN4 trimerization domain (RMKQIEDKIEEIESKQKKIENEIARIKK), and a superfolder green fluorescent protein (GFP) (57) or mOrange2 (58). Mutations in HAs were generated by site-directed mutagenesis. The HAs were purified from cell culture supernatants after expression in HEK293S GnTI(−) cells as described previously (55). In short, transfection was performed using the pCD5 expression vectors and polyethyleneimine I. The transfection mixtures were replaced at 6 h posttransfection by 293 SFM II expression medium (Gibco), supplemented with sodium bicarbonate (3.7 g/L), Primatone RL-UF (3.0 g/L; Kerry, NY, USA), glucose (2.0 g/L), GlutaMAX (1%; Gibco), valproic acid (0.4 g/L), and dimethyl sulfoxide (DMSO) (1.5%). At 5 to 6 days after transfection, tissue culture supernatants were collected, and Strep-Tactin Sepharose beads (IBA, Germany) were used to purify the HA proteins according to the manufacturer’s instructions.

### Glycan microarray binding of HA proteins.

The glycan microarray as earlier presented (17) was utilized. HAs were precomplexed with mouse anti-streptag-horseradish peroxidase (HRP) and goat anti-mouse-Alexa555 antibodies in a 4:2:1 molar ratio respectively in 50 μL phosphate-buffered saline (PBS) with 0.1% Tween 20. The mixture was incubated on ice for 15 min and afterward incubated on the surface of the array for 90 min in a humidified chamber. Then, slides were rinsed successively with PBS-T (0.1% Tween 20), PBS, and deionized water. The arrays were dried by centrifugation and immediately scanned as described previously (17). Processing of the six replicates was performed by removing the highest and lowest replicate and subsequently calculating the mean value and standard deviation over the four remaining replicates. Data Set S1 in the supplemental material presents a full data set of the glycan microarray experiments.

### Hemagglutination assay.

Hemagglutination assays were performed with precomplexed HAs, as described for the glycan microarray, on 1.0% erythrocytes as previously described (55) with a starting concentration of 10 μg/mL of HA for avian H7, H15, and H5 HAs. For equine H7 HAs, a starting concentration of 20 μg/mL HA was used. Erythrocytes were provided by the Department of Equine Sciences and the Department of Farm Animal Health of the Faculty of Veterinary Medicine, Utrecht University, the Netherlands. The blood was taken from adult animals that are in the educational program of the Faculty of Veterinary Medicine. Complete data sets of the hemagglutination assays are present in Data Set S2.

### Protein histochemistry.

Sections of formalin-fixed, paraffin-embedded chicken (Gallus gallus *domesticus*) and equine (Equus ferus *caballus*) trachea were obtained from the Division of Pathology, Department of Biomolecular Health Sciences, Faculty of Veterinary Medicine, Utrecht University, the Netherlands. Tissues from three different horses and chickens were used in the assays to account for biological variation between individuals. In the figures, representative images of at least two individual experiments are shown. Protein histochemistry was performed as previously described (59, 60). In short, tissue sections of 4 μm were deparaffinized and rehydrated, after which antigens were retrieved by heating the slides in 10 mM sodium citrate (pH 6.0) for 10 min. Endogenous peroxidase was inactivated using 1% hydrogen peroxide in methanol for 30 min at room temperature. Tissues were blocked overnight at 4°C using 3% bovine serum albumin (BSA) (wt/vol) in PBS and subsequently stained for 90 min using precomplexed HAs as previously described for the glycan microarray. For avian H7 and H5 HAs, 5 μg/mL HA was used. For H15 HA, we used 2.5 μg/mL HA, and for equine H7 HA, we used 10 μg/mL HA. After washing with PBS, binding was visualized using 3-amino-9-ethylcarbazole (AEC) (Sigma-Aldrich, Germany) and slides were counterstained using hematoxylin.

### Phylogenetic trees.

All available high-quality HA nucleotide sequences (i.e., sequence length is >90% of full-length HA gene segment and has <1% of ambiguous bases) of avian H7Nx and equine H7N7 influenza viruses dated between 1905 and 2005 from the NCBI GenBank database were downloaded (*n* = 944). The maximum-likelihood phylogenetic tree was reconstructed using IQ-TREE (61) by use of the optimal nucleotide substitution model (i.e., GTR+F+R3) based on the Bayesian information criterion as determined by ModelFinder (62). Ancestral sequences were reconstructed using treetime (63).

### Data analysis and statistical analysis.

The data in this article were analyzed and visualized using GraphPad Prism 9.2.0.

### Data availability.

The atomic coordinates and structure factors of the HA of A/Equine/New York/49/73 H7N7 in complex with 3′-GcLN have been deposited in the Protein Data Bank (PDB) under accession code 7T1V.
