# Supplementary material for: N-Glycolylneuraminic Acid Binding of Avian and Equine H7 Influenza A Viruses
Source: J Virol. 2022 Mar 9;96(5):e02120-21. doi: 10.1128/jvi.02120-21 (PMC8906439; doi:10.1128/jvi.02120-21)
Supplement: Supplemental file 1 — Fig. S1 and S2. Download jvi.02120-21-s0002.pdf, PDF file, 0.6 MB [file jvi.02120-21-s0002.pdf]

# Supplementary figures

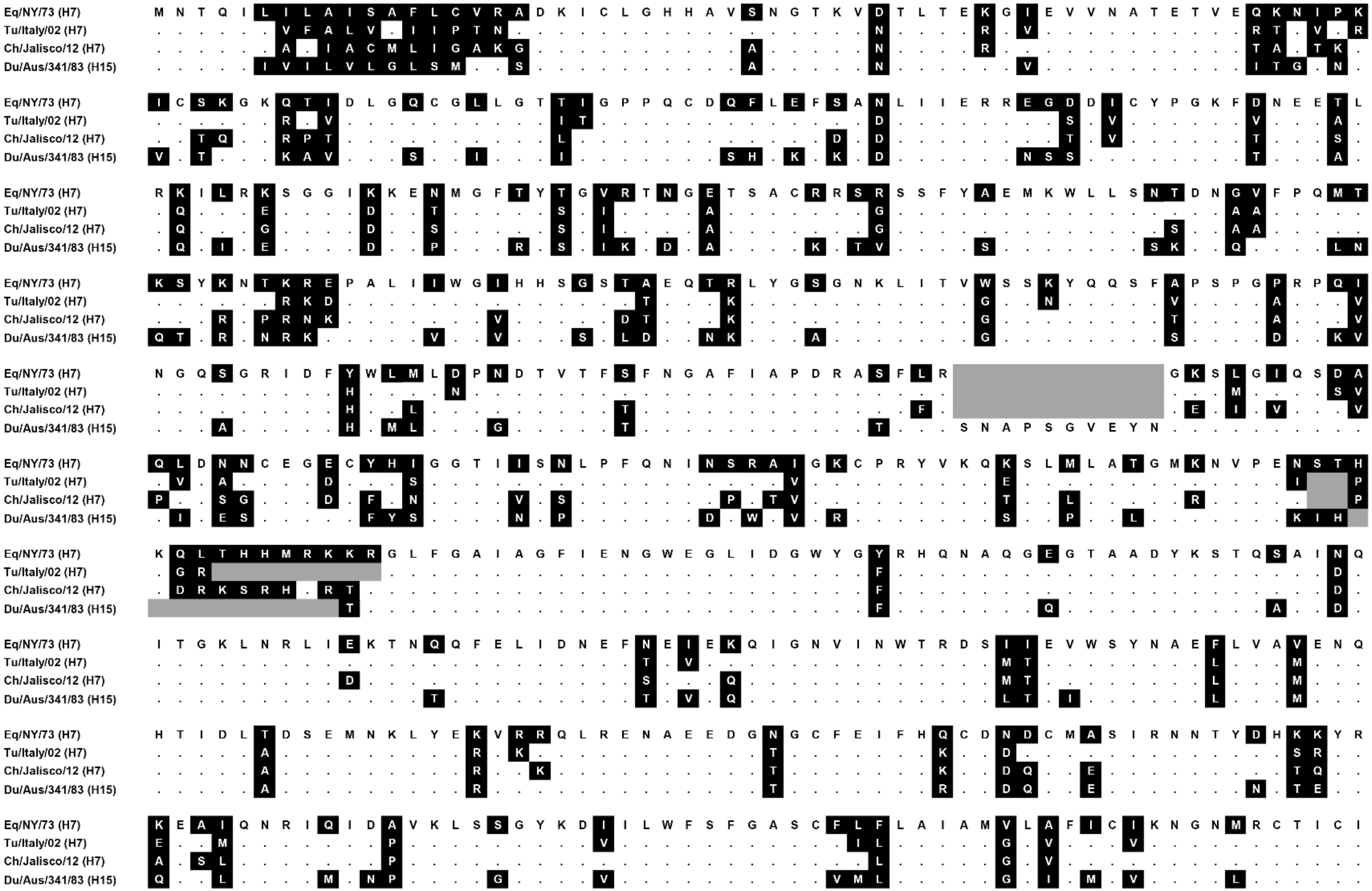

**Fig S1. Amino acid alignment of the HAs of A/Equine/New York/49/73 H7N7, A/Turkey/Italy/214845/02 H7N3, A/Chicken/Jalisco/12283/12 H7N3, and A/Duck/Australia/341/1983 H15N8.** Dots indicate identical amino acids and gray squares indicate deletions.

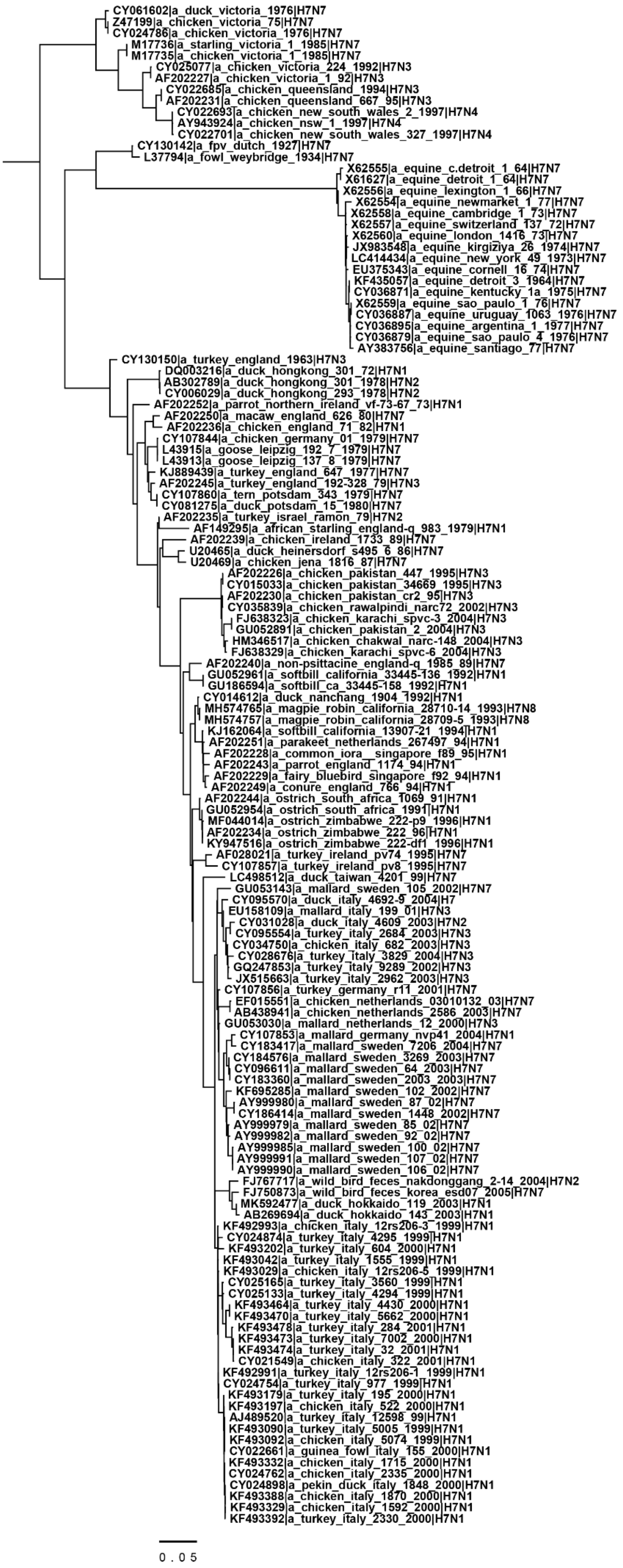

CY061602|a_duck_victoria_1976|H7N7
Z47199|a_chicken_victoria_75|H7N7
CY024786|a_chicken_victoria_1976|H7N7
M17736|a_starling_victoria_1_1985|H7N7
M17735|a_chicken_victoria_1_1985|H7N7
CY025077|a_chicken_victoria_224_1992|H7N3
AF202227|a_chicken_victoria_1_92|H7N3
CY022685|a_chicken_queensland_1994|H7N3
AF202231|a_chicken_queensland_667_95|H7N3
CY022693|a_chicken_new_south_wales_2_1997|H7N4
AY943924|a_chicken_nsw_1_1997|H7N4
CY022701|a_chicken_new_south_wales_327_1997|H7N4
CY130142|a_fpv_dutch_1927|H7N7
L37794|a_fowl_weybridge_1934|H7N7
X62555|a_equine_c.detroit_1_64|H7N7
X61627|a_equine_detroit_1_64|H7N7
X62556|a_equine_lexington_1_66|H7N7
X62554|a_equine_newmarket_1_77|H7N7
X62558|a_equine_cambridge_1_73|H7N7
X62557|a_equine_switzerland_137_72|H7N7
X62560|a_equine_london_1416_73|H7N7
JX983548|a_equine_kirgiziya_26_1974|H7N7
LC414434|a_equine_new_york_49_1973|H7N7
EU375343|a_equine_cornell_16_74|H7N7
KF435057|a_equine_detroit_3_1964|H7N7
CY036871|a_equine_kentucky_1a_1975|H7N7
X62559|a_equine_sao_paulo_1_76|H7N7
CY036887|a_equine_uruguay_1063_1976|H7N7
CY036895|a_equine_argentina_1_1977|H7N7
CY036879|a_equine_sao_paulo_4_1976|H7N7
AY383756|a_equine_santiago_77|H7N7
CY130150|a_turkey_england_1963|H7N3
DQ003216|a_duck_hongkong_301_72|H7N1
AB302789|a_duck_hongkong_301_1978|H7N2
CY006029|a_duck_hongkong_293_1978|H7N2
AF202252|a_parrot_northern_ireland_vf-73-67_73|H7N1
AF202250|a_macaw_england_626_80|H7N7
AF202236|a_chicken_england_71_82|H7N1
CY107844|a_chicken_germany_01_1979|H7N7
L43915|a_goose_leipzig_192_7_1979|H7N7
L43913|a_goose_leipzig_137_8_1979|H7N7
KJ889439|a_turkey_england_647_1977|H7N7
AF202245|a_turkey_england_192-328_79|H7N3
CY107860|a_tern_potsdam_343_1979|H7N7
CY081275|a_duck_potsdam_15_1980|H7N7
AF202235|a_turkey_israel_ramon_79|H7N2
AF149295|a_african_starling_england-q_983_1979|H7N1
AF202239|a_chicken_ireland_1733_89|H7N7
U20465|a_duck_heinersdorf_s495_6_86|H7N7
U20469|a_chicken_jena_1816_87|H7N7
AF202226|a_chicken_pakistan_447_1995|H7N3
CY015033|a_chicken_pakistan_34669_1995|H7N3
AF202230|a_chicken_pakistan_cr2_95|H7N3
CY035839|a_chicken_rawalpindi_narc72_2002|H7N3
FJ638323|a_chicken_karachi_spvc-3_2004|H7N3
GU052891|a_chicken_pakistan_2_2004|H7N3
HM346517|a_chicken_chakwal_narc-148_2004|H7N3
FJ638329|a_chicken_karachi_spvc-6_2004|H7N3
AF202240|a_non-psittacine_england-q_1985_89|H7N7
GU052961|a_softbill_california_33445-136_1992|H7N1
GU186594|a_softbill_ca_33445-158_1992|H7N1
CY014612|a_duck_nanchang_1904_1992|H7N1
MH574765|a_magpie_robin_california_28710-14_1993|H7N8
MH574757|a_magpie_robin_california_28709-5_1993|H7N8
KJ162064|a_softbill_california_13907-21_1994|H7N1
AF202251|a_parakeet_netherlands_267497_94|H7N1
AF202228|a_common_iora__singapore_f89_95|H7N1
AF202243|a_parrot_england_1174_94|H7N1
AF202229|a_fairy_bluebird_singapore_f92_94|H7N1
AF202249|a_conure_england_766_94|H7N1
AF202244|a_ostrich_south_africa_1069_91|H7N1
GU052954|a_ostrich_south_africa_1991|H7N1
MF044014|a_ostrich_zimbabwe_222-p9_1996|H7N1
AF202234|a_ostrich_zimbabwe_222_96|H7N1
KY947516|a_ostrich_zimbabwe_222-df1_1996|H7N1
AF028021|a_turkey_ireland_pv74_1995|H7N7
CY107857|a_turkey_ireland_pv8_1995|H7N7
LC498512|a_duck_taiwan_4201_99|H7N7
GU053143|a_mallard_sweden_105_2002|H7N7
CY095570|a_duck_italy_4692-9_2004|H7
EU158109|a_mallard_italy_199_01|H7N3
CY031028|a_duck_italy_4609_2003|H7N2
CY095554|a_turkey_italy_2684_2003|H7N3
CY034750|a_chicken_italy_682_2003|H7N3
CY028676|a_turkey_italy_3829_2004|H7N3
GQ247853|a_turkey_italy_9289_2002|H7N3
JX515663|a_turkey_italy_2962_2003|H7N3
CY107856|a_turkey_germany_r11_2001|H7N7
EF015551|a_chicken_netherlands_03010132_03|H7N7
AB438941|a_chicken_netherlands_2586_2003|H7N7
GU053030|a_mallard_netherlands_12_2000|H7N3
CY107853|a_mallard_germany_nvp41_2004|H7N1
CY183417|a_mallard_sweden_7206_2004|H7N7
CY184576|a_mallard_sweden_3269_2003|H7N7
CY096611|a_mallard_sweden_64_2003|H7N7
CY183360|a_mallard_sweden_2003_2003|H7N7
KF695285|a_mallard_sweden_102_2002|H7N7
AY999980|a_mallard_sweden_87_02|H7N7
CY186414|a_mallard_sweden_1448_2002|H7N7
AY999979|a_mallard_sweden_85_02|H7N7
AY999982|a_mallard_sweden_92_02|H7N7
AY999985|a_mallard_sweden_100_02|H7N7
AY999991|a_mallard_sweden_107_02|H7N7
AY999990|a_mallard_sweden_106_02|H7N7
FJ767717|a_wild_bird_feces_nakdonggang_2-14_2004|H7N2
FJ750873|a_wild_bird_feces_korea_esd07_2005|H7N7
MK592477|a_duck_hokkaido_119_2003|H7N1
AB269694|a_duck_hokkaido_143_2003|H7N1
KF492993|a_chicken_italy_12rs206-3_1999|H7N1
CY024874|a_turkey_italy_4295_1999|H7N1
KF493202|a_turkey_italy_604_2000|H7N1
KF493042|a_turkey_italy_1555_1999|H7N1
KF493029|a_chicken_italy_12rs206-5_1999|H7N1
CY025165|a_turkey_italy_3560_1999|H7N1
CY025133|a_turkey_italy_4294_1999|H7N1
KF493464|a_turkey_italy_4430_2000|H7N1
KF493470|a_turkey_italy_5662_2000|H7N1
KF493478|a_turkey_italy_284_2001|H7N1
KF493473|a_turkey_italy_7002_2000|H7N1
KF493474|a_turkey_italy_32_2001|H7N1
CY021549|a_chicken_italy_322_2001|H7N1
KF492991|a_turkey_italy_12rs206-1_1999|H7N1
CY024754|a_turkey_italy_977_1999|H7N1
KF493179|a_turkey_italy_195_2000|H7N1
KF493197|a_chicken_italy_522_2000|H7N1
AJ489520|a_turkey_italy_12598_99|H7N1
KF493090|a_turkey_italy_5005_1999|H7N1
KF493092|a_chicken_italy_5074_1999|H7N1
CY022661|a_guinea_fowl_italy_155_2000|H7N1
KF493332|a_chicken_italy_1715_2000|H7N1
CY024762|a_chicken_italy_2335_2000|H7N1
CY024898|a_pekin_duck_italy_1848_2000|H7N1
KF493388|a_chicken_italy_1870_2000|H7N1
KF493329|a_chicken_italy_1592_2000|H7N1
KF493392|a_turkey_italy_2330_2000|H7N1
0.05


6

**Fig S2. Complete annotated phylogenetic tree of equine and Eurasian avian H7 influenza A strains.**

The compact trees that show the variation in amino acids at positions 135, 128, 130, 189, and 193 without strain names are shown in Fig 8A-E.
